# Thermal preference does not align with optimal temperature for aerobic scope in zebrafish (*Danio rerio*)

**DOI:** 10.1242/jeb.243774

**Published:** 2022-11-18

**Authors:** Daniel M. Ripley, Florence A. Quinn, Jessica Dickson, Jack Arthur, Holly A. Shiels

**Affiliations:** ^1^ Division of Cardiovascular Sciences, Faculty of Biology, Medicine and Health, The University of Manchester, Manchester M13 9PL, UK; ^2^ School of Veterinary Medicine and Science, The University of Nottingham, Loughborough LE12 5RD, UK

**Keywords:** Respirometry, OCLTT, Metabolic rate, Temperature preference, Individual variation

## Abstract

Warming is predicted to have negative consequences for fishes by causing a mismatch between oxygen demand and supply, and a consequent reduction in aerobic scope (AS) and performance. This oxygen and capacity limited thermal tolerance (OCLTT) hypothesis features prominently in the literature but remains controversial. Within the OCLTT framework, we hypothesised that fish would select temperatures that maximise their AS, and thus their performance. We tested this hypothesis using intermittent flow respirometry to measure AS at, above (+2.5°C) and below (–2.5°C) the self-selected, preferred temperature (*T*_pref_) of individual zebrafish (*Danio rerio*). AS was greatest 2.5°C above *T*_pref_, which was driven by an increase in maximal metabolic rate. This mismatch between *T*_pref_ and the optimal temperature for AS suggests that factor(s) aside from AS maximisation influence the thermal preference of zebrafish.

## Teaser

**Summary:** Individual zebrafish select temperatures below their optimum for aerobic scope.

## INTRODUCTION

Global temperatures are increasing and will continue rising until at least 2050 (IPCC, 2021). Rising atmospheric temperatures translate to elevated water temperatures, which have profound effects on the metabolism of fishes (Jutfelt, 2020). As fish are ectotherms their body temperature is dictated by their ambient environmental temperature, and so rising environmental temperatures induce concurrent changes in body temperature. Acute changes in body temperature affect the rates of many biological processes, leading to changes in an organism's physiology and biological fitness. In general, acute warming increases biological reaction rates and increases metabolism, whereas cooling slows these processes.

One temperature-dependent trait that has received a lot of interest from the scientific community over the past two decades is aerobic scope (AS) (Pörtner, 2010; Gräns et al., 2014; Clark et al., 2013; Jutfelt et al., 2018; Pörtner et al., 2017; Pörtner, 2014). AS is the difference between an animal's maximum and minimum (standard) metabolic rate and is considered an ecologically relevant metric linked to biological fitness. AS theoretically represents aerobic capacity above an individual's baseline level (i.e. the aerobic capacity that can be utilised for physical activity, digestion, reproduction, etc.; Fry, 1947; Pörtner, 2010). The oxygen and capacity limited thermal tolerance hypothesis (OCLTT) argues that an organism's performance under changing temperatures is dictated by its ability to match tissue oxygen supply and demand (Pörtner, 2010; Pörtner and Farrell, 2008; Pörtner, 2002; Pörtner and Knust, 2007). Within the OCLTT framework, deviation from the temperature at which AS is greatest (temperature for optimum AS, *T*_OptAS_) pushes an animal to its pejus temperatures, resulting in a reduction in physiological performance and biological fitness (Pörtner, 2010; Pörtner and Farrell, 2008; Pörtner, 2002; Pörtner and Knust, 2007). The further the temperature deviates from *T*_OptAS_, the greater the decrease in AS, and the larger the reduction in performance, eventually culminating in an animal reaching its critical thermal limits (CT_max_ and CT_min_), where loss of equilibrium or ‘ecological death’ occurs. Given the central importance of AS within the OCLTT framework, we hypothesised that fish would select the environmental temperature that produces the greatest AS at a given time. This hypothesis has previously been tested in other studies: juvenile barramundi (*Lates calcarifer*) behaviourally select temperatures ∼6°C lower than those that maximise AS (Norin et al., 2014), whilst larval sea lamprey (*Petromyzon marinus*) *T*_pref_ and *T*_OptAS_ roughly align (20.8°C and ∼19.0°C, respectively, Holmes and Lin, 1994). However, these studies did not assess the link between *T*_pref_ and *T*_OptAS_ in individual fish. Here, we test the hypothesis that zebrafish (*Danio rerio*) select a temperature based on AS maximisation by measuring AS at, above, and below the temperature preference (*T*_pref_) of individuals.

## MATERIALS AND METHODS

### Animal husbandry and experimental protocols

**Fig. 1.**
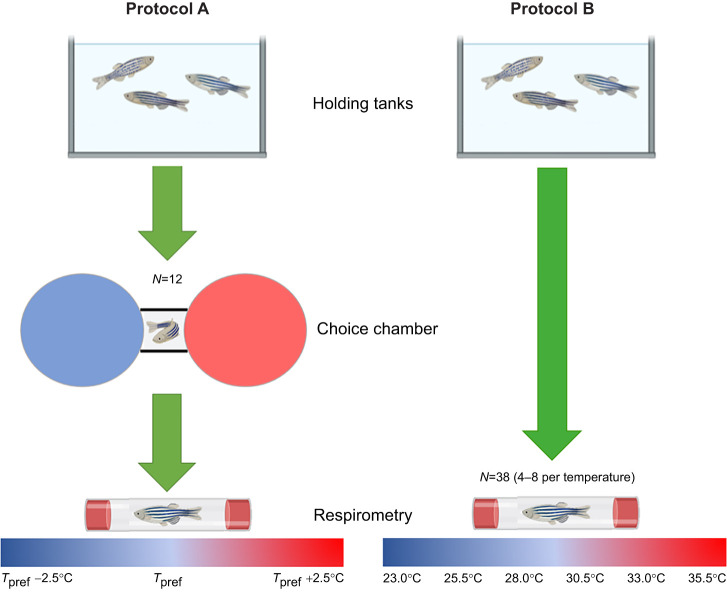
**The experimental setup.** Zebrafish were subject to either protocol A or protocol B. In protocol A, fish underwent a temperature preference (*T*_pref_) trial. Following determination of *T*_pref_, aerobic scope (AS) was measured at the individual's *T*_pref,_ and at 2.5°C above and 2.5°C below *T*_pref_. In protocol B, AS was measured in different fish at 23.0°C, 25.5°C, 28.0°C, 30.5°C, 33.0°C and 35.5°C. Figure created using BioRender.com.

Zebrafish [*Danio rerio* AB strain, mass=0.62±0.019 g (mean±s.e.m.), mixed sex, aged 12–18 months] were raised and maintained at The University of Manchester, UK. Fish were held at 28°C in a 14 h light:10 h dark cycle prior to the experiments. Fish were fasted for 12 h prior to experimentation to achieve a post-absorptive state (confirmed from subsequent oxygen-uptake traces; see doi:10.6084/m9.figshare.19043009). Two protocols (see Fig. 1) were performed in this study and each protocol was performed on a separate group of fish. Separate groups of fish were used for each protocol because of university closure as a result of the Covid-19 pandemic part way through the study. For Protocol A, each fish underwent a choice chamber trial to determine individual *T*_pref_, followed by determination of AS at their *T*_pref_, and at 2.5°C above and 2.5°C below their *T*_pref_. For protocol B, AS was measured at 23.0, 25.5, 28.0, 30.5, 33.0 and 35.5°C to generate a thermal performance curve for zebrafish. Different fish were used at each temperature for the thermal performance curve. Details of each methodology are provided below. All regulated procedures received approval from the institution's ethical review board and were performed under the Home Office License P005EFE9F9 granted to H.A.S.

### Temperature preference

The temperature preference (*T*_pref_) of 13 zebrafish was determined individually using a custom-built shuttlebox system (Fig. S1A). Owing to a technical error, one individual was removed from the final analyses. Two chambers (cylindrical, 20 l each), one at 28°C and one at 30°C, were connected by a transparent acrylic tube (5 cm diameter, 6.5 cm length). Zebrafish were fasted for 12 h prior to the trial. The system was filmed from above, allowing the position of the fish to be tracked throughout the duration of the trial (1 h acclimation to shuttlebox system followed by 4 h of data collection). Once the trial started, the fish was able to alter the temperature of the system based on its location. When the fish was in the warmer chamber, the entire system would heat at 4°C h^−1^. When the fish was in the cooler chamber, the system would cool at 4°C h^−1^. Thus, the fish was able to alter its environmental temperature by selecting either the colder or warmer chamber. Heating and chilling were achieved by pumping water from hot (50°C) or cold (4°C) reservoirs through titanium plates under the base of the experimental chambers. A stick heater was present in each chamber and, when required, was manually used to maintain the 2°C difference between chambers if any drift occurred. A re-circulating pump in each chamber mitigated thermal stratification and was positioned to limit mixing between the warm and cold chambers. Temperature in each chamber was measured every 30 s using an Elitech RC-4 temperature data logger (±0.05°C) (Elitech Technology, Inc., Milpitas, CA, USA) (Fig. S1B). The final *T*_pref_ was taken as the mean temperature of the chambers occupied by the fish throughout the duration of the trial, excluding the acclimation period (Fig. S2, Table S1). After the *T*_pref_ trial, fish were transferred to holding tanks at 28°C for a minimum of 24 h. Fish were fed whilst in the holding tanks, but fasted for 12 h before the assessment of AS.

### Assessment of aerobic scope by respirometry

Respirometry trials were performed to assess maximal metabolic rate (MMR) and standard metabolic rate (SMR) to calculate AS. For protocol A, three respirometry trials were performed on the individual zebrafish (*N*=12) used in the *T*_pref_ trials. Each fish had one trial at its *T*_pref_, one 2.5°C above its *T*_pref_ and one 2.5°C below its *T*_pref_ [*T*_pref_=28.63±0.53°C (mean±s.e.m.); range, 25.55–31.42°C; *N*=12; Table S1]. The fish was taken up/down to the appropriate temperature at a rate of 2.5°C h^−1^ prior to AS measurement, which occurred as soon as the test temperature was reached. Trial order was randomised, and a minimum of 24 h left between trials to allow for recovery. The trial order for individual fish was determined using a random number generator.

For protocol B, MMR, SMR and AS were estimated through the measurement of oxygen uptake rates for zebrafish at 23.0°C, 25.5°C, 28°C, 30.5°C, 33.0°C and 35.5°C to create temperature performance curves. To do this, 28°C acclimated zebrafish were transferred to a 1 litre tank and the temperature raised/dropped from 28°C to the test temperature at a rate of 2.5°C h^−1^. Different fish were used at each temperature in protocol B to mitigate training effects and cumulative stress.

Oxygen uptake rate was measured using a custom-built intermittent flow respirometry system. Four 100 ml (including the volume of associated tubing) Plexiglass chambers were submerged in a 60 l header tank that was supplied with temperature controlled (±0.3°C), aerated, filtered (200 µm filter) water from a 40 l buffer tank. Water was mixed within the chambers through a re-circulating loop where oxygen content was measured every second (4-channel Firesting oxygen logger, Pyroscience). Each respirometry loop consisted of a 60 s flush phase, 120 s wait period and 300 s measurement phase. During the flushing phase, water from the buffer tank was pumped into the chambers (40 l h^−1^ chamber^−1^) to re-oxygenate the system and prevent the waste products of metabolism accumulating. In the wait period, the chamber was functionally sealed so the oxygen content of the water declined linearly based on the rate at which the fish extracted oxygen. Finally, during the measurement phase, the linear decline in oxygen content over time was measured and used to calculate the fish's aerobic metabolic rate. AquaResp 3.0 was used for the automation of aquatic respirometry (University of Copenhagen; www.aquaresp.com).

To elicit MMR, fish were chased for 180 s (cylindrical tank, 100 ml of water) prior to being transferred through the air (standardised to 30 s) and placed in the respirometry chamber during the wait phase. Chasing typically occurred in the afternoon, following which the fish were left in the dark until the following morning to reach standard metabolic rate (SMR). Owing to the relatively long wait and measurement phases (420 s combined), MMR is likely to be underestimated in our study; however, this underestimation is probably consistent between our groups and accordingly should not influence our conclusions. Prior to, and immediately following each experiment, background respiration in the system was measured (over 1500 s). Following each experiment, the fish were returned to their holding tank and the respirometry system was bleached, drained, rinsed and re-filled for subsequent trials.

### Data analysis

FishResp v.1.0.4 (Morozov et al., 2019) in R v.4.1.2 (https://www.r-project.org/) was used to process the respirometry data. MMR was calculated as the steepest slope (*r*^2^ ≥0.95) recorded in the first hour of recordings following the chase. SMR was calculated as the lowest 10% of values (*r*^2^≥0.90) recorded throughout the remaining portion of the trial. MMR and SMR were background corrected using the linear relationship between background respiration and time to subtract an appropriately weighted background respiration measurement from each oxygen uptake measurement. Background respiration (mean±s.e.m) accounted for 18.6±0.91% and 40.8±2.19% of uncorrected MMR and SMR, respectively. AS was calculated as MMR–SMR using the background respiration corrected data.

The relationships between mean SMR, MMR and AS against temperature were modelled in GraphPad Prism v.9.1.0 with second order polynomial (SMR; *r*^2^=0.87, MMR; *r*^2^=0.93) and Gaussian (AS; *r*^2^=0.79) functions, respectively. AS, SMR and MMR were compared across temperatures using one-way ANOVAs in Prism.

SMR, MMR and AS when measured 2.5°C above, 2.5°C below and at each individual's *T*_pref_ were compared using lme4 (https://CRAN.R-project.org/package=lme4) and lmerTest (https://CRAN.R-project.org/package=lmerTest) mixed effects models in R v.4.1.2 (Bates et al., 2015; Kuznetsova et al., 2017). Temperature group (at, above, or below *T*_pref_) and trial order were included as fixed effects, and individual included as a random effect. The residuals from each model conformed to normal distributions (Shapiro–Wilk tests; *P*>0.05), and pairwise comparisons between temperature groups were computed using emmeans (https://CRAN.R-project.org/package=emmeans) in R v.4.1.2.

## RESULTS AND DISCUSSION

**Fig. 2.**
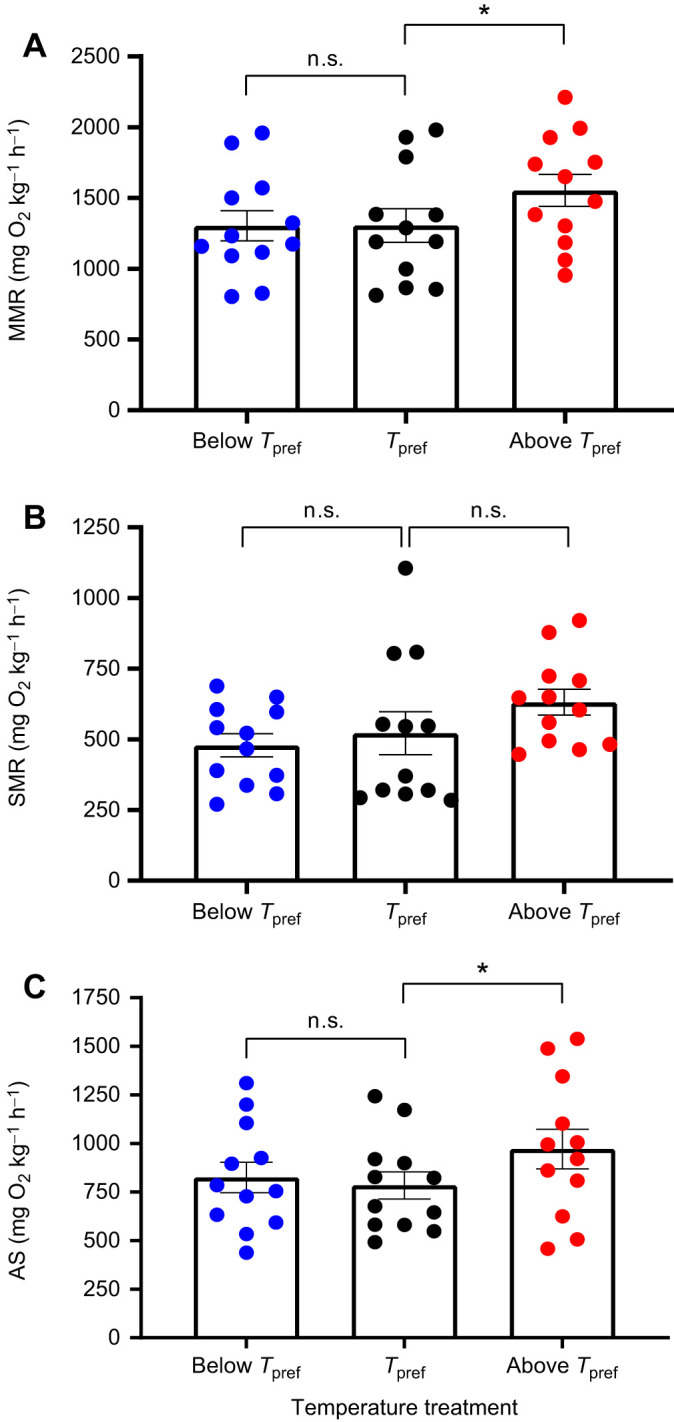
**Maximum metabolic rate, standard metabolic rate and aerobic scope of zebrafish (*Danio rerio*) measured at their individual temperature preference (*T*_pref_) and at 2.5°C above and 2.5°C below *T*_pref_.** (A) maximum metabolic rate (MMR), (B) standard metabolic rate (SMR) and (C) aerobic scope (AS) of zebrafish measured at *T*_pref_ (black), and at 2.5°C above (red) and 2.5°C below (blue) *T*_pref_. Bars show the mean±s.e.m. Individual data points are overlaid as dots. Mixed effects model, pairwise comparisons computed using emmeans. *P<0.05; *N*=12; n.s., not significant.

Here, we show that the preferred temperature of individual zebrafish [*T*_pref_ =28.63±0.53°C (mean±s.e.m.), range 25.55–31.42°C, *N*=12; Table S1], which is comparable to previous estimates (Rey et al., 2015), is below the temperature at which their aerobic scope (AS) is greatest (*T*_OptAS_) (*P*=0.031, Fig. 2C). An increase in AS 2.5°C above mean *T*_pref_ was driven by a significant increase in MMR (*P*=0.0048, Fig. 2A*)*, but there was no change in SMR (*P*=0.13, Fig. 2B). AS, MMR and SMR did not differ between *T*_pref_ and 2.5°C below *T*_pref_ (AS, *P*=0.40, Fig. 2C; MMR, *P*=0.81, Fig. 2A; SMR, *P*=0.45, Fig. 2B). Previous studies on juvenile baramundi support this mismatch between *T*_pref_ and *T*_OptAS_ (Norin et al., 2014, but c.f*.* Holmes and Lin, 1994). However, to our knowledge, this is the first study to directly demonstrate that *T*_pref_ and *T*_OptAS_ are not consistent within the same animal, at the same age, under the same conditions.

The oxygen and capacity limited thermal tolerance (OCLTT) hypothesis emphasises a key role for AS in the physiology of aquatic ectotherms (Pörtner and Farrell, 2008; Pörtner, 2010). OCLTT proposes that as environmental temperatures move away from an optimum, AS decreases and a mismatch between tissue oxygen demand and supply limits the performance of the animal. Within this framework, it can be expected that an animal would choose to inhabit environmental conditions that maximise AS and thus performance and fitness. However, we show that the *T*_pref_ of zebrafish is misaligned with the temperature at which their AS is greatest, such that individual fish have a greater AS 2.5°C above their *T*_pref_ than at their *T*_pref_. This mismatch between *T*_pref_ and *T*_OptAS_ implies that AS is not the sole determinant of performance during temperature changes. The *T*_Opt_ for various performance metrics in ectotherms are known to differ. *T*_Opt_ for growth and AS in Atlantic halibut (*Hippoglossus hippoglossus*) are known to be misaligned (Gräns et al., 2014), as are the *T*_Opt_ for AS and escape velocity in spiny lobster (*Sagmariasus verreauxi*) larvae, but not juveniles (Twiname et al., 2020). Given that *T*_Opt_ differs between biological processes, the mismatch between *T*_OptAS_ and *T*_pref_ in our study suggests that the thermal conditions selected by individual zebrafish are not solely governed by the temperature performance curve of AS, but by the thermal performance curves of other traits or combinations of traits.

The multiple performances multiple optima (MPMO) hypothesis, like OCLTT, recognises that AS is a temperature-dependent trait that contributes to an organism's biological fitness (Clark et al., 2013). However, unlike OCLTT, MPMO treats AS as one of several physiological parameters that dictate performance across temperatures, and states that the optimal temperature for each of these parameters need not be the same (Clark et al., 2013). Our data show that zebrafish choose to inhabit temperatures below their *T*_OptAS_, implying that traits other than AS dictate the preferred, and thus likely optimal, temperature of zebrafish. Previous research supports this conclusion, with a study on juvenile barramundi (*Lates calcarifer*) measuring *T*_pref_ as 31.7°C, but showing an increase in AS up to 38°C (Norin et al., 2014).

**Fig. 3.**
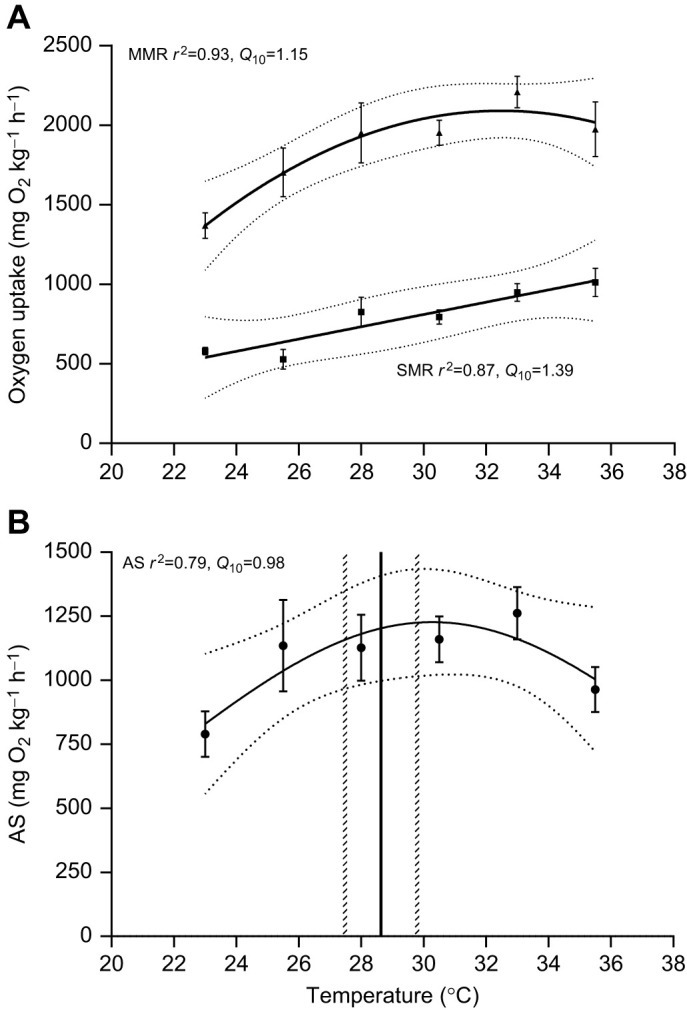
**Relationship between temperature and maximum metabolic rate, standard metabolic rate and aerobic scope in zebrafish.** (A) SMR (squares) and MMR (triangles), and (B) AS. Data shown are means±s.e.m. Solid lines represent the fitted curves, with the 95% confidence intervals plotted as horizontal dashed grey lines. Vertical black line shows the mean temperature preference, with the 95% confidence intervals plotted as vertical dashed grey lines. *N*=4–8 per temperature (*N*=8 for 33.0°C; *N*=7 for 23.0°C and 30.5°C; *N*=6 for 25.5°C and 28.0°C, and *N*=4 for 35.5°C) for SMR, MMR and AS, and *N*=12 for temperature preference (*T*_pref_).

Despite AS increasing at 2.5°C above *T*_pref_ in individual zebrafish, at the ‘population level’ (here defined as different groups of fish used at each temperature), there was no significant relationship between AS and temperature (AS; *P*=0.07, tested between 23.0 and 35.5°C) owing to increasing MMR and SMR (SMR, *P*<0.0001; MMR, *P*=0.0005) with similar *Q*_10_ values (MMR *Q*_10_=1.15; SMR *Q*_10_=1.39). Superficially, this lack of relationship between AS and temperature appears to contradict the observation that AS is more than 2.5°C above *T*_pref_ rather than at *T*_pref_. However, considering the intraspecific variation in *T*_pref_ measured in this study (range 25.55–31.42°C, *N*=12), temperatures that were clos­­e to *T*_pref_ for some individuals would be below, or above, the *T*_pref_ of others. This variation in *T*_pref_ and likely *T*_OptAS_ may preclude any population-level relationship between temperature and AS because, across any given temperature change, the AS of individuals within the population would be changing in different ways. Warming waters may therefore be favouring certain individuals within a population, even when population-level effects of warming are not apparent. Zebrafish have a broad *T*_OptAS_ (Fig. 3B), as is characteristic of eurytherms. Future studies should test whether more stenothermal species, which may display a clearer and narrower *T*_OptAS_ (Farrell, 2016), show this same disparity between individual- and population-level changes in AS across a range of temperatures.

### Conclusion

Oceans and rivers are warming at an unprecedented rate. A recent IPCC report suggests that under all probable emissions scenarios, global surface temperatures will continue to rise until at least the middle of the 21st century, with temperature increases expected to exceed 1.5°C (IPCC, 2021). As water temperatures rise, it is critical to empirically test current theories and hypotheses to develop a more thorough physiological understanding of temperature's effects on fishes. Here, we demonstrate that individual zebrafish select thermal conditions that do not maximise their aerobic scope, but that this effect is masked at the population-level by intraspecific variability. These data highlight the need to consider individuals within a population, as well as populations as a whole, when investigating fish physiology under climate change.

## Supplementary Material

10.1242/jexbio.243774_sup1 Supplementary information
