# Correction: Thermal preference does not align with optimal temperature for aerobic scope in zebrafish (*Danio rerio*)

**DOI:** 10.1242/jeb.245488

**Published:** 2023-01-26

**Authors:** Daniel M. Ripley, Florence A. Quinn, Jessica Dickson, Jack Arthur, Holly A. Shiels

There was an error in *J. Exp. Biol.* (2022) **225**, jeb243774 (doi:10.1242/jeb.243774).

The *Q*_10_ values presented in Results and Discussion and in Fig. 3 are incorrect, as a result of a formulaic error. Original *Q*_10_ values: AS *Q*_10_=0.98, SMR *Q*_10_=1.39, MMR *Q*_10_=1.15; corrected *Q*_10_ values: AS *Q*_10_=1.17, SMR *Q*_10_=1.56, MMR *Q*_10_=1.34. The corrected and original versions of Fig. 3 are shown below.

Both the online full text and PDF versions of the paper have been corrected. The authors apologise to the readers for this error, which does not impact the statistical analysis, interpretation or conclusions of the paper.

**Fig. 3 (corrected).**
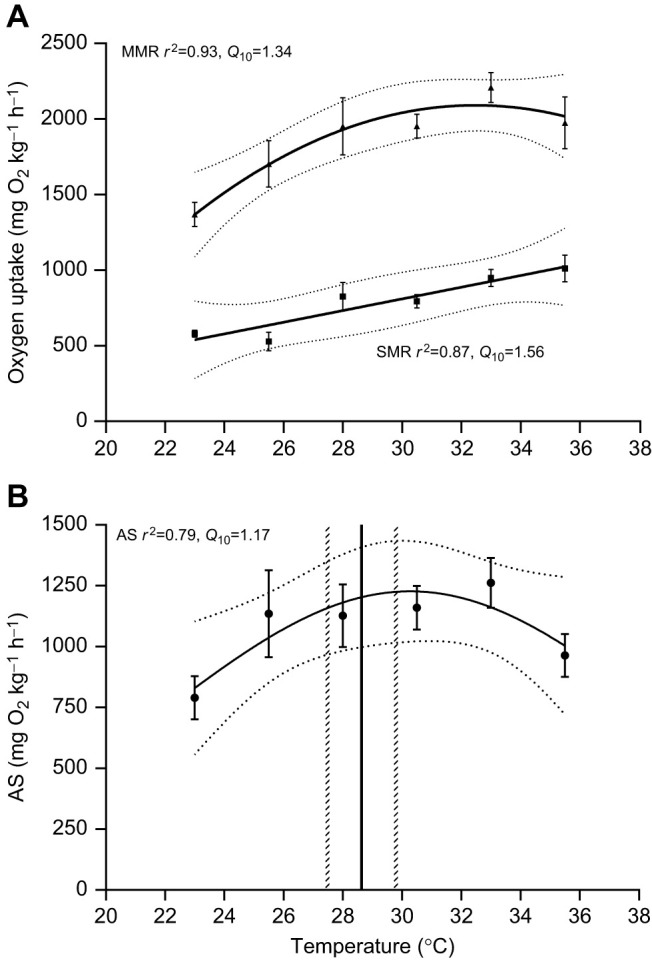
**Relationship between temperature and maximum metabolic rate, standard metabolic rate and aerobic scope in zebrafish.** (A) SMR (squares) and MMR (triangles), and (B) AS. Data shown are means±s.e.m. Solid lines represent the fitted curves, with the 95% confidence intervals plotted as horizontal dashed grey lines. Vertical black line shows the mean temperature preference, with the 95% confidence intervals plotted as vertical dashed grey lines. *N*=4–8 per temperature (*N*=8 for 33.0°C; *N*=7 for 23.0°C and 30.5°C; *N*=6 for 25.5°C and 28.0°C, and *N*=4 for 35.5°C) for SMR, MMR and AS, and *N*=12 for temperature preference (*T*_pref_).

**Fig. 3 (original).**
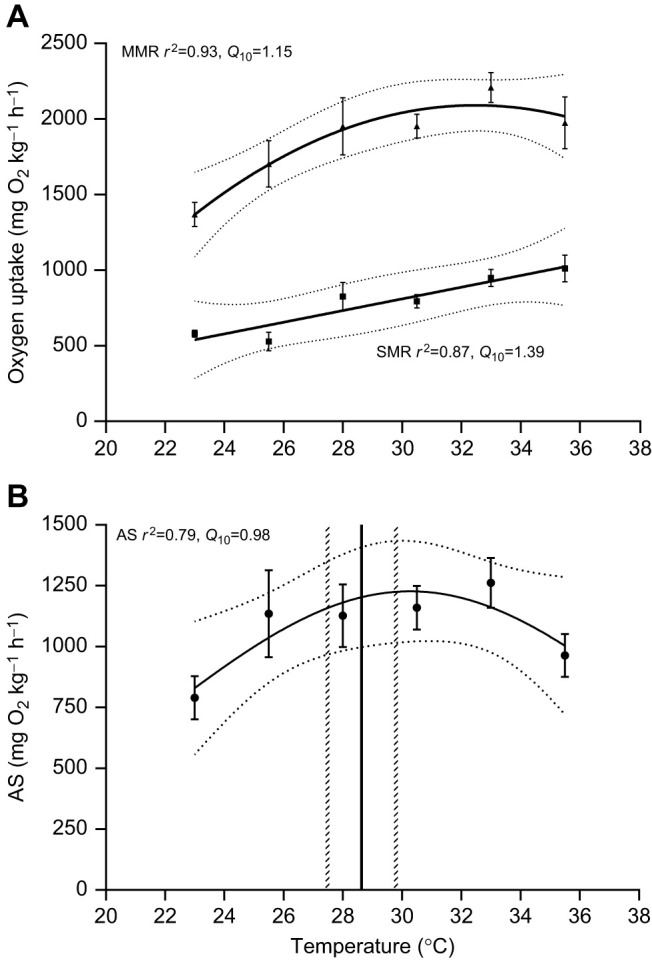
**Relationship between temperature and maximum metabolic rate, standard metabolic rate and aerobic scope in zebrafish.** (A) SMR (squares) and MMR (triangles), and (B) AS. Data shown are means±s.e.m. Solid lines represent the fitted curves, with the 95% confidence intervals plotted as horizontal dashed grey lines. Vertical black line shows the mean temperature preference, with the 95% confidence intervals plotted as vertical dashed grey lines. *N*=4–8 per temperature (*N*=8 for 33.0°C; *N*=7 for 23.0°C and 30.5°C; *N*=6 for 25.5°C and 28.0°C, and *N*=4 for 35.5°C) for SMR, MMR and AS, and *N*=12 for temperature preference (*T*_pref_).

