# Supplementary material for: Thermal preference does not align with optimal temperature for aerobic scope in zebrafish (Danio rerio)
Source: J Exp Biol. 2022 Nov 18;225(22):jeb243774. doi: 10.1242/jeb.243774 (PMC9845742; doi:10.1242/jeb.243774)
Supplement: Supplementary information [file jexbio-225-243774-s1.pdf]

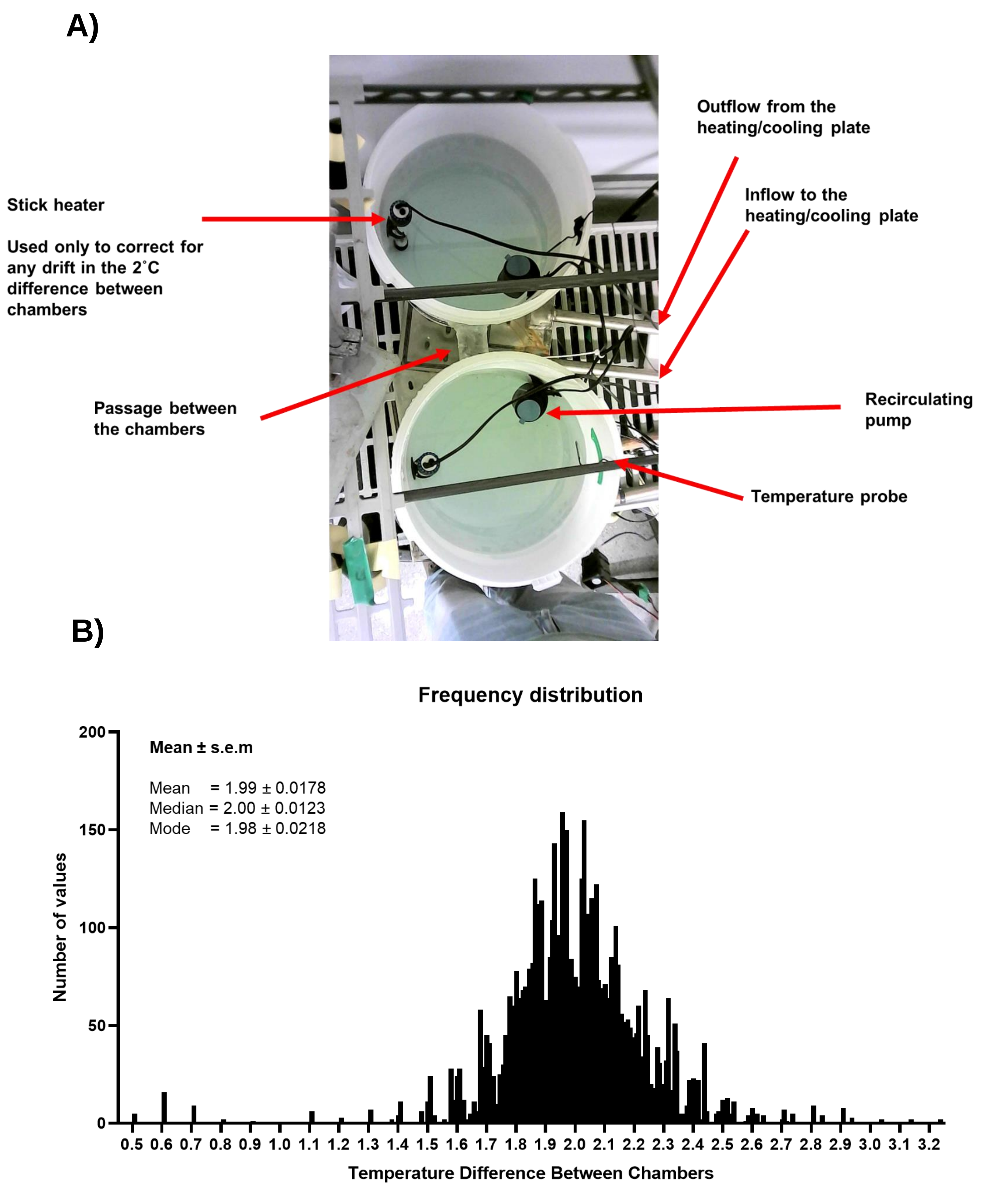

Fig. S1. A) A labelled photograph of the choice chambers and **B)** a histogram showing the distribution of the temperature difference between the two choice chambers. A temperature reading for each chamber was taken once every 30 seconds. The average (mean, median, and modal) temperature differences are shown in the inset. N = 12 trials.

Journal of Experimental Biology • Supplementary information

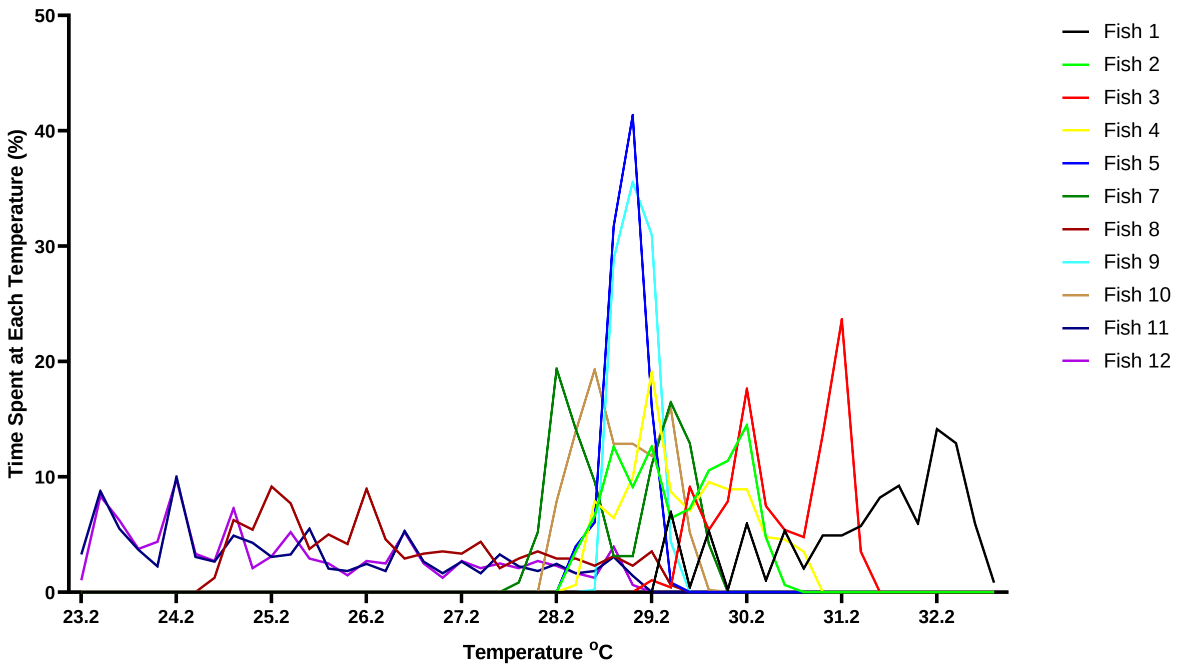

Fig. S2. Line plot showing the percentage of time each fish spent at each temperature during the temperature preference experiment. N = 12.

Table S1. Mean, median, and modal temperature preference of each zebrafish.

Fish	Mean T_{pref}	Median T_{pref}	Modal T_{pref}
1	31.42	31.70	32.50
2	29.48	29.45	29.95
3	30.56	30.55	31.15
4	29.54	29.40	29.10
5	28.94	28.95	29.00
6	29.12	29.00	28.93
7	28.90	28.78	28.25
8	26.6	26.3	24.95
9	29.03	29.00	29.05
10	28.89	28.88	29.03
11	25.55	25.40	23.00
12	25.58	25.40	24.40
Mean ± s.e.m	28.63 ± 0.53	28.57 ± 0.56	28.20 ± 0.81

Journal of Experimental Biology • Supplementary information
